# Avascular osteonecrosis of the jaw following orthognathic surgery: a systematic review

**DOI:** 10.3389/froh.2026.1826043

**Published:** 2026-06-23

**Authors:** Chaoqun Huang, Feng Zhang, Weixu Jin, Liwei Zhou, Gary Shun Pan Cheung, Wang-yong Zhu

**Affiliations:** 1 Department of Dental Surgery, The University of Hong Kong-Shenzhen Hospital, Shenzhen, Guangdong, China; 2 Shenzhen Clinical Research Center for Rare Diseases, Shenzhen, China

**Keywords:** avascular necrosis, avascular osteonecrosis of the jaw, ischemic complication, Le Fort I osteotomy, orthognathic surgery

## Abstract

Orthognathic surgery plays a crucial role in restoring occlusal function and improving facial aesthetics. Avascular osteonecrosis of the jaw is a rare but severe postoperative complication. Here, we reviewed the clinical characteristics and management of avascular osteonecrosis of the jaw following orthognathic surgery. A systematic review of published studies up to April 2026 was conducted. Data on clinical features, potential contributing factors, and management strategies were collected. This review included 17 studies encompassing 33 cases. Avascular osteonecrosis of the jaw was predominantly located in the maxilla (28/33), with the majority of cases (18/33) affecting the alveolar bone of the maxilla. Early symptoms primarily included gingival or soft tissue discoloration (14/33). As necrosis progressed, symptoms such as tooth loss, alveolar bone loss, oronasal fistulas, and others were observed. Management followed a staged approach: non-surgical interventions for initial control and surgical interventions for progressive cases. Thirteen cases ultimately underwent bone grafting and flap reconstruction. Larger sample sizes and higher-quality studies are needed to further understand this rare but serious complication.

## 1 Introduction

Orthognathic surgery plays a critical role in restoring occlusal function and enhancing facial aesthetics (1–3). As its use expands, complications such as intraoperative or postoperative hemorrhage, iatrogenic fracture, infection, delayed bone union or nonunion, and neurologic injury are increasingly recognized (4). Among these, avascular osteonecrosis of the jaw is a severe but rare postoperative complication, with an incidence of 0.3% (5). The morbidity associated with vascular compromise or ischemic complications varies with maxillary or mandibular procedures (6). Various studies have proposed potential etiologies for avascular osteonecrosis of the jaw following orthognathic surgery, including intraoperative vascular injury, palatal mucosal tears, multi-segment surgery, anatomical factors, and systemic factors (3, 7–9). Although infrequent, this complication is irreversible and can have devastating consequences once it occurs (3, 10). To minimize this risk, it is recommended to assess ischemic risk factors, provide thorough patient counseling, select appropriate surgical plans preoperatively, implement preventive measures, and meticulous intraoperative manipulation to avoid iatrogenic vascular injury (3, 7, 8). To our knowledge, this is the first systematic review focusing on avascular osteonecrosis of the jaw following orthognathic surgery. In this review, we summarize the associated risk factors and therapeutic strategies for this rare but severe complication, aiming to provide a comprehensive clinical review to enhance understanding and management.

## 2 Methods

### 2.1 Search strategy

A systematic literature search was conducted in the PubMed, Embase, Scopus, and Cochrane databases for articles published before April 2026. The search strategy was presented as follows:

[(maxill*) OR (mandib*) OR (jaw)] AND [(osteotomy) OR (orthognathic) OR (lefort) OR (genioplasty) OR (cleft) OR (mandibular angle) OR (ramus) OR (subapical)] AND [(avascular) OR (ischemic) OR (necrosis) OR (aseptic)].

### 2.2 Selection of studies

Articles were initially screened based on the title and abstract. Then full texts were reviewed to confirm eligibility.

The inclusion criteria were defined as follows:

(1) Studies should be clinical trials, including randomized controlled trials, cohort studies, case-control studies, and case reports.
(2) Publications should report cases of jaw osteonecrosis following orthognathic surgery.
(3) Publications should provide sufficient clinical information and detailed management strategies for jaw osteonecrosis in orthognathic surgery.

The exclusion criteria include studies without English translations.

All studies were independently reviewed by two authors. In cases of disagreement, a third author was brought in to review the studies, and any inconsistencies were resolved through discussion.

### 2.3 Data extraction and analysis

The extracted data included patient sex and age, the extent of the lesion prior to treatment, the predominant anatomical location of the lesion (maxilla or mandible), type of surgery performed, occurrence of vascular injury, potential contributing factors (anatomic, surgical, and systemic), previous medical history, postoperative infection, management strategies, and reconstruction methods.

### 2.4 Assessment of risk of bias in included studies

The assessment of bias was conducted using the Methodological Index for Non-Randomized Studies (MINORS) criteria. The bias scale had a maximum score of 16 (11).

### 2.5 Statistical analysis

Descriptive statistics were used to analyze data regarding the type of osteotomy, vascular injury, segmental surgery, management, and reconstruction.

## 3 Results

### 3.1 Results of the search

**Figure 1:**
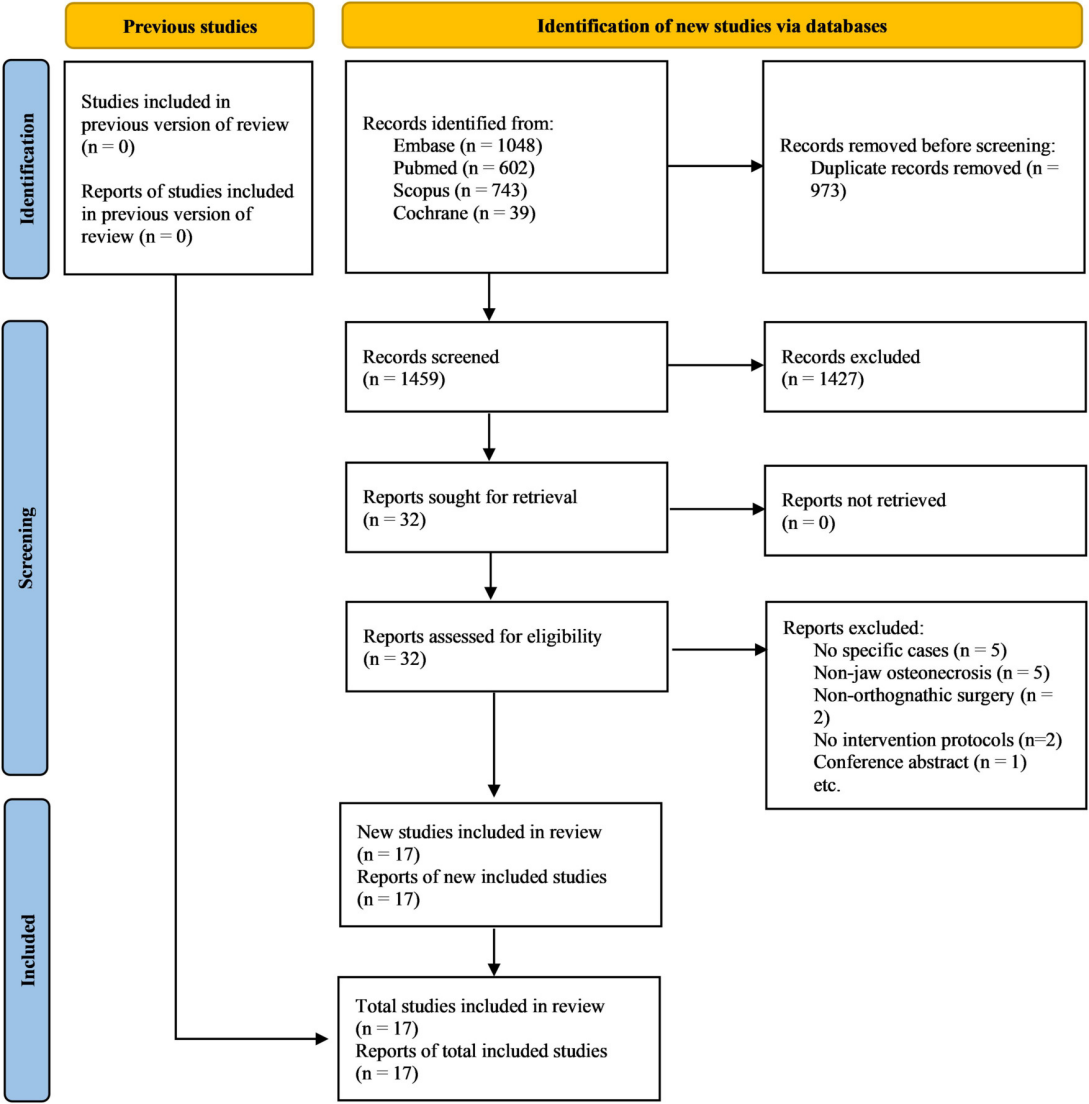
PRISMA flowchart of literature selection (From database inception to 2026 April).

602 articles were identified in PubMed, 1,048 in Embase, 743 in Scopus, and 39 in the Cochrane Library. After removing of duplicate articles, 1,459 unique articles remained. Based on the title and abstract screening, 32 articles were selected for full text review. Ultimately, 17 articles were included in the systematic review (Figure 1). All included studies were retrospective studies or case reports.

### 3.2 Risk of bias of included studies

The Supplementary Table provides an assessment of the risk of bias for the studies included in the systematic review, based on the MINORS criteria.

### 3.3 Description of patient demographics

Demographic data from the included studies are presented in Table 1 (2, 3, 6–10, 12–21). This systematic review encompasses a total of 33 patients, of whom seven were male, 24 were female, and the sex of two patients was not reported. The mean age, reported for 31 patients, was 31 years, with a range of 13 to 51 years.

**Table 1:** Demographic characteristics of the included studies.

NO.	Author & year	No. of patients	Age (Year)	Sex	Surgical procedures	Location of avascular osteonecrosis	Clinical manifestations	Management	Follow-up
1	Kato, H.et al. (12).	1	25	F	LF I and BSSRO	Right anterior maxillary alveolar bone	Maxillary alveolar bone exposure, oronasal regurgitation, osseous non-union of the maxilla and tooth mobility	Initial saline irrigation of sequestra, debridement of necrotic tissue, extraction of relevant teeth, iliac crest grafting, tooth extraction, endodontic treatment, iliac crest bone grafting, implants prosthodontics, oral vestibular extension operation	5 years after reconstruction
2	Nezafati, S. et al. (13).	1	35	F	LF I and Genioplasty	Fistula in incisive papilla	Oronasal regurgitation, bad odor and taste along with a slight change in voice	Von-langenbeck flap for closing the fistula	6 months after reconstruction
3	Lanigan, D. T. et al. (6).	15	32.1 (19–48)	M, *n* = 4;	Segmented LF I, *n* = 8	Maxillary alveolar bone, labial bone and palatal region	Slough of gingivae, discoloration of gingivae and mucosa, necrosis of the periodontal tissues, teeth nonvital, alveolar bone loss, oronasal fistulas and maxillary defect	Endodontic treatment, tooth extraction, HBOT, antibiotic therapy, fistula closure, maxillary and palatal reconstruction, and implant prosthodontics	Average 11.2 months (3–18)
				F, *n* = 11					
					LF I, *n* = 1				
					Segmented LF I, BSSRO and Genioplasty, *n* = 1				
					Segmented LF I and BSSRO, *n* = 1				
					Segmented LF I and Genioplasty, *n* = 1				
					Wassmund Anterior Maxillary Osteotomy, *n* = 1				
					Left Hemi LF I and BSSRO, *n* = 1				
					Anterior Maxillary Osteotomy, *n* = 1				
4	Lanigan, D. T. et al. (8).	2	1: 38	F	1: BSSRO	1: mandibular vertical ramus;	1: infection, exposure of right superior border intraosseous wire, slight suppurative discharge, lack of secondary wound healing and bone exposure remained	1: HBOT (20 sessions), doxycycline and debridement	1: 9 years
					2: BSSRO and Genioplasty				2: N/A
							2: infection, detachment of the geniohyoid muscles;	2: intravenous antibiotics; removal of proplast implant and necrotic bone	
			2: 20			2: mental region			
5	Mercuri, L. G. et al. (14).	1	14	F	BSSRO and Genioplasty	Anterior portion of lower mandibular border free graft	Infection	Antibiotics and debridement	6 months
6	Heggie, A. et al. (9).	1	22	M	Segmented LF I	Maxillary alveolar bone surrounding central incisors, canines	Bone exposure of bone limited to the cleft side and tooth loss	Removal of central incisors and sequestrating labial plate; canine removal; on-lay grafting and implants prosthodontics	17 months
7	Behnia, H. et al. (15).	1	40	F	Premaxillary Osteotomy	Premaxilla	N/A	Two-stage modiﬁed prefabricated free ﬁbula ﬂap reconstruction and implants prosthodontics	11 years after reconstruction
8	Le, J. M. et al. (16).	1	51	F	LF I	Nearly complete maxilla	Posterior oronasal fistula, exposure of maxillary bone, tooth mobility necrosis of soft tissue, gingival recession (2–3 mm), mobility of the alveolar segment	Local wound care, antibiotics, chlorhexidine gluconate irrigation, HBOT, and pentoxifylline and tocopherol, staged surgical debridement, and reconstruction using three-segment FVFF, implants prosthodontics and patient-specific selective laser melted custom plate	8 months
9	Ettinger, K. S. et al. (3).	1	20	F	LF I, BSSRO and Genioplasty	Total maxilla	N/A	Debridement, fibular free flap reconstruction, and implants prosthodontics	2.5 years after reconstruction
10	Parnes, E. I. et al. (17).	1	13	M	Premaxillary Osteotomy	Anterior maxilla	Loss of alveolar bone, exposure of roots of UR1 and UR2; failure of the gingival pedicle graft	Removal of necrotic teeth and sequestrum, debridement and temporary denture	3 months
11	Alalawy, H. et al. (18).	1	27	F	BSSRO	Partial right mandibular angle	Numbness of lower lip on the right side	Left side: correcting the deviation of mandible by vertical ramus subsigmoid osteotomy	2 years after reconstruction
								Right side: first stage: reconstruction plate and bone grafting; second stage: maxillary and chin advancement; third stage: LF I advancement, alveolar cleft defect bone graft augmentation, and genioplasty	
12	Kim, S. et al. (19).	1	37	F	Lefort I and Transoral Vertical Ramus Osteotomy	Partial right mandibular proximal segment	Infection	Antibiotics and surgical debridement	3 months
13	Teemul, T. A. et al. (17).	1	45	F	Segmented LF I	Right maxilla	Infection and oroantral fistula in the upper right quadrant	Antibiotics, HBOT, pentoxifylline and Vitamin E	6 months
14	Moran, I. et al. (20).	2	N/A	N/A	LF I	1: anterior maxilla;	1: tooth no vitality and mobility;	1: surgical debridement, iliac crest bone grafting and implants prosthodontics	N/A
						2: bony dehiscence associated UL1		2: tailored oral hygiene instruction, surgical debridement of the alveolar bone, and soft tissue grafting.	
							2: isolated gingival necrosis around the UL1 and an associated bony dehiscence		
15	Singh, J. et al. (10).	1	15	F	LF I, BSSRO and Genioplasty	Nearly total maxillary alveolar bone	Right maxillary sinusitis, tooth mobility, transverse alveolar collapse of the maxilla, mandibular anterior posterior excess and asymmetry, chin deviation to the right, occlusal instability, difficulty in mastication, maxillary pain, pronounced facial asymmetry and malocclusion	HBOT, debridement, bone grafting, implants prosthodontics	15 months after reconstruction
16	Yeo, J. F. et al. (21).	1	22	M	LF I	Torus palatinus	Gingival recession, labial crestal bone loss involving tooth 13, right buccal mucosal pedicle laceration and temporary complete loss of sensitivity of the palatal mucosa	Antibiotics, corticosteroids, salicylate, and pentoxifylline	8 weeks
17	Holm, C. K. et al. (2).	1	43	F	Segmented LF I and BSSRO	Maxillary vestibular alveolar bone	Infection, necrosis of the vestibular gingival tissue in the UR3-UL3 region, oronasal fistula in the palate, teeth 13 and 23 no vitality, recurrent bilateral maxillary sinusitis, sensory dysfunction of the left mandible	Antibiotics, endodontic treatment, tooth extraction, debridement, local vascularized buccal flap and local palatal flap used to close the fistula, fixed dental bridges	4 years

F, female; LF I, lefort I osteotomy; BSSRO, bilateral sagittal split ramus osteotomy; M, male; HBOT, hyperbaric oxygen therapy; N/A, not available; FVFF, free vascularized fibula flap; UR, upper right; UL, upper left.

### 3.4 Surgical information

Among all cases, there were 11 instances of bimaxillary surgery, including 6 cases of Le Fort I osteotomy (Le Fort I) combined with Bilateral Sagittal Split Ramus Osteotomy (BSSRO), 2 cases of Le Fort I combined with BSSRO and genioplasty, 2 cases of Le Fort I combined with genioplasty, and 1 case of Le Fort I combined with Vertical Ramus Osteotomy. Additionally, 18 cases underwent isolated maxillary osteotomy, comprising 14 cases of Le Fort I, and 4 cases of premaxillary osteotomy. Four cases received isolated mandibular osteotomy, including 2 cases of BSSRO and 2 cases of BSSRO combined with genioplasty.

Among the 29 cases that underwent maxillary surgery, 25 cases involved Lefort I. Of these, 14 cases underwent segmental Le Fort I osteotomy, including 4 cases of 2-segment Le Fort I, 4 cases of 3-segment Le Fort I, 4 cases of 4-segment Le Fort I, and 2 cases where the number of segments was not specified. Additionally, 4 cases underwent premaxillary osteotomy.

Vascular injuries were reported in 9 of the 33 cases. The most common vascular injury was ligation of the descending palatine artery (DPA) (*n* = 6). The other three vascular injuries included injury to the left external carotid artery, injury to the right inferior alveolar artery, and cauterization of the nasopalatine artery.

### 3.5 Medical history

Five patients had a medical history of cleft lip and palate. Additionally, one patient had a medical history of sickle cell disease (SCD) and a fibro-osseous lesion presenting as a hyperplastic right maxillary tuberosity.

### 3.6 Location of avascular osteonecrosis

The anatomical location of avascular osteonecrosis of the jaw was reported in all 33 patients, with 28 cases occurring in the maxilla and 5 cases in the mandible. Eighteen cases involved only the alveolar bone, whereas 15 cases affected the body of the maxilla or mandible. Within the maxilla, common sites included the alveolar bone (*n* = 18) surrounding specific teeth, such as the central incisors, canines, and premolars, or on the labial side; the anterior maxilla (*n* = 4); the palatal region (*n* = 3); and the body of the maxilla (*n* = 3). In the mandible, osteonecrosis was observed in the mandibular vertical ramus, the chin bony segment, and the lower mandibular border.

### 3.7 Clinical manifestations

At the onset of avascular osteonecrosis of the jaw, several clinical manifestations may be observed. The primary symptom was a change in the color of the gingiva or surrounding soft tissues (*n* = 14), including blackening, cyanosis, pallor, or a bluish-gray hue, typically detectable immediately after surgery or within one week. Other manifestations included soft tissue necrosis and sloughing (*n* = 6) and gingival recession (*n* = 1). In the surgical area, wound dehiscence (*n* = 1), purulent discharge (*n* = 1), swelling (*n* = 3), pain (*n* = 1), and fistula formation (*n* = 1) were also identified. Radiographically, early signs included widening of the periodontal ligament space (*n* = 1). Additionally, symptoms such as premature anterior occlusal contact, mandibular deviation, and numbness of the lower lip were reported in one case.

As necrosis progressed, additional clinical manifestations were documented, including loss of tooth vitality, tooth mobility, and sloughing of teeth within or adjacent to the necrotic area (*n* = 19); alveolar bone loss (*n* = 20); and oronasal fistulas resulting in nasal leakage during swallowing in cases involving the palate (*n* = 9). Furthermore, secondary complications such as maxillary sinusitis (*n* = 2), secondary malocclusion (*n* = 1), facial asymmetry (*n* = 1), and poor healing of fracture sites (*n* = 2) were also observed.

### 3.8 Managements

The interval between the onset of these symptoms and the initial surgical intervention varies from 2 weeks to 8 years, while the interval from symptom onset to reconstructive repair spans from 3 months to 8 years.

Twelve patients underwent antibiotic treatment with agents such as penicillin, tetracycline, erythromycin, doxycycline, clindamycin, third-generation cephalosporins, metronidazole, amoxicillin-clavulanate, benzylpenicillin, and phenoxymethylpenicillin. Additional medications administered were pentoxifylline (*n* = 3), vitamin E (*n* = 2), corticosteroids (*n* = 1), and salicylates (*n* = 1).

Initial non-surgical interventions included hyperbaric oxygen therapy (HBOT) (*n* = 11), with the number of sessions ranging from 2 to 24, irrigation (*n* = 2), tailored oral hygiene instruction (*n* = 1), local wound care (*n* = 1), and partial denture restoration (*n* = 2).

As the symptoms progressed, further interventions were undertaken, including debridement (*n* = 17), tooth extraction (*n* = 10), endodontic treatment (*n* = 3), bone grafting (*n* = 7), flap reconstruction (*n* = 8), and other procedures such as implant prosthodontics (*n* = 7), skin graft vestibuloplasty (*n* = 1), and soft tissue grafting (*n* = 1).

Thirteen cases underwent bone grafting and/or flap reconstruction. Six patients received bone grafts harvested from the iliac crest, with graft sites including the anterior maxillary alveolar bone (*n* = 2), premaxilla (*n* = 2), palate (*n* = 1), and mandible (*n* = 1). One patient underwent onlay bone grafting, while the source of the graft material was not specified. Three patients received free vascularized fibular flap reconstructions, involving the total maxilla (*n* = 2) and partial maxilla (*n* = 1). Four patients underwent local flap procedures, comprising a von Langenbeck flap for closure of an incisive papilla fistula (*n* = 1), buccal flaps for closure of an oronasal fistula (*n* = 1) and a fistula of the maxillary vestibule (*n* = 1), as well as a combination of rotated palatal flap, mucosal flap from the lip, and tongue flap (*n* = 1). Table 2 presents the major interventions, ranging from conservative non-surgical therapies to advanced free vascularized flap reconstructive surgeries.

**Table 2:** Summary of interventions.

Main Interventions	Subtype of Intervention	Details & Case Number (n)
Non-surgical interventions	Systemic antimicrobials	*n* = 12; penicillin, tetracycline, erythromycin, doxycycline, clindamycin, third-generation cephalosporins, metronidazole, amoxicillin-clavulanate, benzylpenicillin, and phenoxymethylpenicillin
	Adjunctive pharmacotherapy	Pentoxifylline (*n* = 3), vitamin E (*n* = 2), corticosteroids (*n* = 1), and salicylates (*n* = 1)
	Hyperbaric oxygen therapy	*n* = 11
	Other non-surgical interventions	Irrigation (*n* = 2), tailored oral hygiene instruction (*n* = 1), local wound care (*n* = 1), and partial denture restoration (*n* = 2)
Surgical interventions	Debridement	*n* = 17
	Tooth extraction	*n* = 10
	Bone grafting	*n* = 6
	Flap reconstruction	Local flaps (*n* = 4), free vascularized fibular flap (*n* = 3)
	Implant prosthodontics	*n* = 7
	Other surgical interventions	Endodontic treatment (*n* = 3), skin graft vestibuloplasty (*n* = 1), soft tissue grafting (*n* = 1)

## 4 Discussion

This study provides a systematic review of the risk factors, clinical manifestations, and management strategies related to avascular osteonecrosis of the jaw following orthognathic surgery. To our knowledge, this represents the first systematic review addressing avascular osteonecrosis of the jaw following orthognathic surgery.

Factors associated with avascular osteonecrosis of the maxilla have been reported to include ligation of the DPA during maxillary surgery, potential narrowing of the DPA resulting from previous palatal procedures (e.g., surgeries in patients with cleft lip and palate), and diminished blood supply due to excessive surgical segmentation (8).

Several studies have recognized ligation of the DPA as one of risk factors for jaw necrosis following maxillary osteotomy (2, 3, 7, 8, 13). Preservation of the DPA during Le Fort I osteotomy is justified by its critical role in maintaining blood supply and nutritional support to the anatomical region, thereby optimizing maxillary integrity and reducing the risk of avascular necrosis (8). Conversely, some studies have reported that DPA ligation does not significantly affect average maxillary gingival blood flow (22). Experimental animal studies have further demonstrated that although DPA ligation causes a transient reduction in blood supply to the mobilized bone segment, no significant difference in blood supply is observed one week postoperatively (23). Additionally, the final severity of ischemic injury depends not only on the resistance of various jaw tissue cells to ischemia, but also on the capacity of blood vessels to restore and maintain perfusion in the affected area. A substantial proportion of such injuries may be more accurately described as “post-ischemic reperfusion injury” (24). Selective preservation of the DPA and minimization of its injury can be achieved by retracting the DPA with a specialized retractor (25), releasing the pyramidal process of the palatine bone (26, 27), or utilizing an ultrasonic bone knife to protect the DPA during bone removal (12). Clinical evidence indicates that the vascular supply to the mobilized maxilla depends on a rich, anastomotic vascular network of multiple arterial sources, and that the patency of the DPA alone does not guarantee bone viability. Maxillary osteonecrosis may still develop despite the presence of an intact and patent DPA (3). Additional factors, such as the integrity of the palatal soft tissue pedicle (3, 7, 9), the extent of surgical dissection (10), and the quality of intraoperative handling (7), may also play critical roles in maintaining adequate perfusion and preventing postoperative complications.

In patients with cleft palate, the incidence of postoperative maxillary avascular necrosis is higher compared to individuals without cleft palate (9). The impairment of the palatal tissues may increase the risk of maxillary avascular necrosis, likely due to scarring from previous cleft lip and palate surgeries, which results in a reduced vascular supply to the mucoperiosteal tissues (28). A study of 12 cleft palate patients demonstrated that preoperative arteriographic analysis revealed a clinical reduction in the size of 10 out of 24 DPAs, suggesting that primary and/or revisional palatal surgeries may adversely affect the overall blood supply to the palatal pedicle (29). Therefore, a thorough preoperative evaluation of palatal tissue scarring and the number of previous surgeries is essential; selective carotid angiography may be used to assess the arterial blood supply to the maxilla. Intraoperatively, it is imperative to stretch tissues gradually and cautiously to avoid rapid tearing. Additionally, simultaneous maxillary advancement and mandibular setback should be considered to “share the correction load” (28, 29).

Avascular necrosis of the maxilla is more commonly observed in the anterior region following segmental osteotomy. To minimize this risk, it is recommended to limit the division of the maxilla to as few bone segments as possible, especially avoiding small anterior segments (28). Preoperative orthodontic treatment should be optimized to minimize the number of osteotomy segments required. In cases involving significant transverse discrepancies planned for bimaxillary surgery, mandibular arch narrowing via median symphyseal osteotomy may be considered as an alternative to performing all transverse corrections exclusively on the maxilla (8).

Avascular necrosis following mandibular orthognathic surgery is extremely rare (18, 19). This low incidence is attributed to the mandible's rich blood supply and robust collateral circulation, as well as the meticulous preservation of periosteal and soft tissue perfusion during surgical procedures (6). Nevertheless, in patients undergoing BSSRO, a short and thin mandibular ramus has been identified as a risk factor for osteonecrosis (30). This increased risk arises because extensive periosteal dissection is often required to avoid intraoperative fractures caused by damage to the bone contact area during fixation of bone segments, thereby increasing the likelihood of bone ischemia and subsequent osteonecrosis (18, 31). Unscrutinized soft tissue dissection of the short mandibular ramus may result in the stripping of a considerable surface area of bone from its musculo-periosteal coverage, disrupting the majority of its blood supply (18). In addition, non-rigid fixation can promote movement of bone segments at the osteotomy or fracture site, adversely affecting revascularization and bone healing. It is suggested that reducing the dissection of soft tissues from the proximal segment and minimizing myo-periosteal dissection to improve bone perfusion and reduce postoperative bone loss (32).

Systemic factors that increase the risk of avascular necrosis of the jaw include SCD. Trevor et al. reported a case involving a patient with SCD who underwent a Le Fort I osteotomy combined with a right posterior segmental osteotomy, subsequently developed wound infection and maxillary ischemia one week postoperatively (7). For patients with SCD or those at risk of severe hypoxia, prophylactic blood transfusion to maintain hemoglobin S levels below 30% is recommended to reduce the incidence of complications (33).

Vascular damage should be assessed by evaluating gingival cyanosis and congestion intraoperatively or postoperatively. If persistent cyanosis is observed in the attached gingiva, release of fixation and/or loosening of the suspension should be performed. Additionally, the palate must be examined for accidental entrapment of the palatal pedicle and/or stretching of the palatal mucoperiosteum. After excluding palatal pedicle stripping or transection, blood perfusion can be restored (28). If gingival cyanosis persists after correcting peri-anesthetic hypotension and fixing the osteotomized segment in its new position, indicating that the viability of the osteotomized segment may be compromised, the segment should be repositioned to its original location, properly fixed, and the surgical procedure discontinued (34). In cases where severe tissue perfusion issues are identified immediately postoperatively, the patient should be promptly returned to the operating room for repositioning of the osteotomized segment (10).

There is minimal overlap between the signs and symptoms of avascular osteonecrosis of the jaw, with few significant clinical signs and symptoms suggestive of the extensive underlying process (6). Objective signs, including gingival discoloration, cyanosis, mucosal sloughing, bone exposure, and fistula formation, appear early, often within days after surgery. In contrast, subjective symptoms such as pain, nasal regurgitation, numbness, malocclusion, and difficulty eating are mild or absent in the early stages and usually develop later, coinciding with progressive bone and soft tissue destruction. Only pain, swelling, and fistula-related nasal leakage show slight overlap between signs and symptoms. These findings suggest that early diagnosis depends primarily on identifying clinical signs rather than patient-reported symptoms.

In cases of established avascular osteonecrosis of the jaw, it is recommended to maintain good oral hygiene, perform conservative debridement, and HBOT 20 to 30 sessions (8, 35, 36). Reconstruction of hard and soft tissue defects is generally postponed (34). Patients may undertake careful local irrigation using sterile normal saline and/or diluted hydrogen peroxide independently. When clinical signs of infection, microbial culture and antimicrobial susceptibility testing should be conducted, followed by the administration of appropriate systemic antibiotics to control the infection. In such instances, extraction of the affected teeth is initially advised to preserve bone tissue and prevent more severe complications. Early surgical removal of the affected bone segment is considered only if the infection persists (28). HBOT should be implemented as early as possible, as it may accelerate the demarcation of the necrotic area and its extent; however, it cannot reverse the progression of avascular necrosis once established, although it may limit its severity (8). If the necrotic bone remains intact and is firmly stabilized, partial revascularization often occurs, which helps prevent significant osseous defects and the formation of oral-nasal and/or oral-antral fistulas (28).

In patients presenting with a well-defined boundary between the jaw lesion and the surrounding bone tissue, or a clear demarcation, including cases where necrotic bone is separated from healthy bone by soft tissue mucosa, surgical intervention can be effectively performed by removing the sequestrum alone. Surgical treatment involves thorough resection of necrotic bone tissue until healthy, vascularized bone margins are exposed, avoiding residual necrotic foci; the bone margins should be smoothed intraoperatively to prevent secondary injury to soft tissues. The wound is subsequently closed using tension-free sutures to ensure adequate mucosal coverage and to facilitate optimal healing (37).

Reconstruction utilizing local or pedicle flaps, as well as free soft tissue flaps, is effective for fistula closure, defect repair, and coverage of exposed bone tissue (38). The free fibula flap (FFF), widely employed in clinical practice for mandibular reconstruction, is beneficial for implant-supported rehabilitation following jaw reconstruction (39). The application of computer-assisted surgery enhances the precision of osteotomy, improves the predictability of the final reconstruction outcome, and reduces intraoperative duration (40). The modified prefabricated free fibula flap (PFFF), which is pre-cultured in the patient's leg for a period to promote flap maturation prior to transplantation, can avoid the defect of flap tissue bulkiness, is more suitable for maxillary reconstruction, and facilitates subsequent denture rehabilitation (15).

Several limitations should be acknowledged in our study. First, all included studies were retrospective studies or case reports, with no randomized controlled trials available. such selection bias may have compromised the reliability of the findings. Second, the included studies featured small sample sizes and incomplete data, which hindered the validation of the significance of various factors and limited the strength of the evidence supporting the effectiveness of the treatment recommendations. Third, complete follow-up data were unavailable for some patients, complicating the systematic analysis of follow-up duration.

## 5 Conclusion

This systematic review, which includes 17 studies, analyzed the clinical characteristics of avascular osteonecrosis of the jaw following orthognathic surgery. It synthesized potential etiologies of the condition, as well as both initial and delayed management strategies. Larger number of sample sizes and higher-quality studies are necessary to enhance the understanding of this rare but serious complication.

## Data Availability

Datasets will be available by request. Requests to access these datasets should be directed to Wang-yong Zhu, zhuwy1@hku-szh.org.
