# Supplementary material for: Avascular osteonecrosis of the jaw following orthognathic surgery: a systematic review
Source: Front Oral Health. 2026 Jun 23;7:1826043. doi: 10.3389/froh.2026.1826043 (PMC13337813; doi:10.3389/froh.2026.1826043)
Supplement: Supplementary file 1 [file Table1.docx]

**Supplementary Table.** The risk bias assessment for the included studies in the systematic review.

| **Study** | **A clearly stated aim** | **Inclusion of consecutive patients** | **Prospective collection of data** | **Endpoints appropriate to the aim of the study** | **Unbiased assessment of the study endpoint** | **Follow-up period appropriate to the aim of the study** | **Loss to follow up less than 5%** | **Prospective calculation of the study size** | **Total scores (maximum 16)** |
| --- | --- | --- | --- | --- | --- | --- | --- | --- | --- |
| Kato, H.et al. 2020. | 2 | 0 | 0 | 2 | 2 | 2 | 2 | 0 | 10 |
| Nezafati, S. et al. 2023. | 2 | 0 | 0 | 2 | 2 | 2 | 2 | 0 | 10 |
| Lanigan, D. T. et al. 1990. | 2 | 0 | 0 | 2 | 2 | 2 | 0 | 0 | 8 |
| Lanigan, D. T. et al. 1990. | 2 | 0 | 0 | 2 | 2 | 2 | 0 | 0 | 8 |
| Mercuri, L. G. et al. 1977. | 2 | 0 | 0 | 2 | 2 | 2 | 2 | 0 | 10 |
| Heggie, A. et al. 2021. | 2 | 0 | 0 | 2 | 2 | 2 | 2 | 0 | 10 |
| Behnia, H. et al. 2009. | 2 | 0 | 0 | 2 | 2 | 2 | 2 | 0 | 10 |
| Le, J. M. et al. 2022. | 2 | 0 | 0 | 2 | 2 | 2 | 2 | 0 | 10 |
| Ettinger, K. S. et al. 2020. | 2 | 0 | 0 | 2 | 2 | 2 | 2 | 0 | 10 |
| Parnes, E. I. et al. 1972. | 2 | 0 | 0 | 2 | 2 | 2 | 2 | 0 | 10 |
| Alalawy, H. et al. 2022. | 2 | 0 | 0 | 2 | 2 | 2 | 2 | 0 | 10 |
| Kim, S. et al. 2014. | 2 | 0 | 0 | 2 | 2 | 2 | 2 | 0 | 10 |
| Teemul, T. A. et al. 2017. | 2 | 0 | 0 | 2 | 2 | 2 | 2 | 0 | 10 |
| Moran, I. et al. 2018. | 2 | 1 | 0 | 2 | 2 | 2 | 2 | 0 | 11 |
| Singh, J. et al. 2008. | 2 | 0 | 0 | 2 | 2 | 2 | 2 | 0 | 10 |
| Yeo, J. F. et al. 1989. | 2 | 0 | 0 | 2 | 2 | 2 | 2 | 0 | 10 |
| Holm, C. K. et al. 2023. | 2 | 0 | 0 | 2 | 2 | 2 | 2 | 0 | 10 |

The Methodological Index for Non-Randomized (MINORS) criteria were used to assess the risk of bias in included studies. The items are scored 0 (not reported), 1 (reported but inadequate) or 2 (reported and adequate).
